# Public Health-Led Insights on Electric Micro-mobility Adoption and Use: a Scoping Review

**DOI:** 10.1007/s11524-023-00731-0

**Published:** 2023-05-16

**Authors:** Alexandra Bretones, Oriol Marquet, Carolyn Daher, Laura Hidalgo, Mark Nieuwenhuijsen, Carme Miralles-Guasch, Natalie Mueller

**Affiliations:** 1 Research Group On Mobility, Transportation and Territory (GEMOTT), Geography Department, Autonomous University of Barcelona (UAB), Barcelona, Spain; 2 Institute of Environmental Science and Technology (ICTA), Autonomous University of Barcelona (UAB), Barcelona, Spain; 3 ISGlobal, Barcelona, Spain; 4 Universitat Pompeu Fabra (UPF), Barcelona, Spain; 5 CIBER Epidemiología Y Salud Pública (CIBERESP), Madrid, Spain

**Keywords:** Electric micro-mobility, e-Scooters, e-Bikes, Determinants, Public health, Scoping review

## Abstract

The advent of electric micro-mobility (EMM) has transformed the urban mobility landscape, with projections indicating a 5–10% increase in its modal share in European cities by 2030. In this scoping review, we aimed to comprehensively examine the key determinants of EMM adoption and usage from a public health perspective. Sixty-seven articles were included in the analysis, primarily covering e-bikes and e-scooters. The determinants were categorised into two broad categories: (1) contextual determinants that encompass enabling and hindering factors related to legal frameworks, transportation systems and infrastructure, and technology, and (2) individual-level determinants that pertain to intrinsic motivations and deterrents of individuals. Our findings reveal that EMM vehicles are widely perceived as a cost-effective, flexible, ad hoc, and fast mode of transportation within urban areas, augmenting accessibility and connectivity. Additionally, the lightweight, foldable, and transportable nature of these vehicles is highly appreciated by users. However, several barriers have also been identified, including inadequate infrastructure and end-of-trip facilities, limited capability to traverse diverse terrains and trip scenarios, acquisition and maintenance costs, limited carrying capacities, technical failures, and accident risks. Our results suggest that the interplay of contextual enablers and barriers and personal motivations and deterrents drive the emergence, adoption, and usage of EMM. Hence, a comprehensive understanding of both contextual and individual-level determinants is crucial for ensuring a sustainable and healthy uptake of EMM.

## Introduction

Electric micro-mobility (EMM) is emerging as a transformative transport mode in cities globally, filling a previously undefined niche in terms of its users, opportunities, risks, and impacts. As cities strive towards a sustainable and zero-carbon future, the consideration of these new modes of transport becomes increasingly crucial in discussions surrounding urban mobility and environmental sustainability. For instance, EMM is believed to have the potential to reduce greenhouse gas (GHG) emissions, air pollution, and congestion [1–3], while increasing accessibility and connectivity, and facilitating first- and last-mile mobility [4–8] [4–7, 9]. Moreover, the user travel experience while riding may be enhanced, as these new modes can provide a more engaging experience with the travel environment, a joyful alternative to getting around, and even impact health and well-being outcomes [10–12].

EMM includes a range of small-sized, lightweight, electrically powered vehicles that, typically, facilitate short trips of up to 10 km, and thereby extend the distances users can travel without a car [13, 14]. In European settings, EMM vehicles can usually carry one or two passengers, and sometimes cargo, operate at low speeds (i.e. up to 25 km/h), but sometimes up to moderate speeds (i.e. up to 45 km/h) [15], and they can be privately owned or accessed through sharing systems. Vehicles that commonly meet the rather broad EMM definition are e-bikes, e-trikes or e-cargo bikes, and various forms of e-scooters and e-rickshaws, but also one-wheeled, two (or more)-wheeled balancing boards, including e-skateboards and Segways. The classification of EMM is a complex issue, in part due to the lack of consistent legal definitions across European countries and local jurisdictions. Therefore, EMM taxonomy and classifications have usually been defined by the combination of two of the following elements: vehicle weight, vehicle maximum speed, and vehicle capacity However, this conventional approach has been challenged by authors such as Christoforou et al.[16], who propose a more mobility-oriented definition that considers EMM to encompass all modes of transportation that enable users to seamlessly transition between pedestrian and vehicular modes as necessary. In this scoping review, we follow Christoforou et al.[16] understanding of EMM. We are excluding larger and more powerful vehicles, such as e-mopeds and e-motorcycles.

These new e-powered micro-vehicles are gaining popularity in cities worldwide, and a 5–10% increase in EMM modal share is expected by 2030 in the European Region [17]. With this rise in popularity, it is important to understand the determinants of EMM use, i.e. what enables and motivates users, and what barriers and deterrents they encounter. Current research shows how EMM can provide individuals with an accessible, relatively cheap and fast way to move around [18, 19], increase accessibility and connectivity for certain groups, and have important equity implications in terms of transport choices and associated health and well-being outcomes. According to various studies, EMM perceived benefits include convenience, freedom, flexibility, and overcoming car dependence [20, 21]; provision of exercise [22–24]; enabling mobility for users with physical limitations [12, 25]; reduced travel time [16, 26, 27]; economic savings [16, 27]; respect for the environment [23, 27]; fun, enjoyability, and enhanced human experience [16, 24]; and general contribution to increased well-being [24].

At the same time, certain deterrents and barriers have been also identified such as safety concerns [27–30]; lack of appropriate infrastructure, poor road conditions, and lack of end-of-trip facilities [24, 28, 29, 31]; traffic noise and air pollution [32]; vehicle acquisition and maintenance costs [33, 34]; limited carrying capacity [21, 35]; fear of theft and vandalism [12, 36, 37]; and fear of technical weaknesses and failure [12, 24].

These positive and negative determinants might vary widely according to contextual settings, transport needs, habits and patterns, individual perceptions, and previous experiences. Thus, given the predicted increase in EMM usage in urban settings and the acknowledged relationship between modal choice and health, it is necessary to include a public health viewpoint to better understand the impact of EMM on the health and well-being of individuals and communities, as the use of EMM can have a significant impact on public health outcomes, for instance on physical activity, air and noise pollution, safety and accessibility, among others. Understanding the factors that influence EMM adoption can help identify and address barriers and promote its adoption as a healthy and sustainable transportation option. Furthermore, understanding the deterrents can help in the design and implementation of policies and infrastructure that can mitigate these barriers and increase safety for all.

This scoping review summarises the existing literary landscape on the determinants of EMM use and adoption from a public health perspective, to help European authorities better understand EMM patterns and user behaviours.

## Methods

A scoping literature review was selected as the most appropriate method for research objectives. In contrast with a systematic review—a comprehensive and rigorous method of reviewing the literature on a specific research question, following a predefined and systematic process to identify, appraise, and synthesise all relevant studies on a topic—scoping reviews are a useful tool for assessing the breadth and focus of a body of literature on a specific topic, providing an overview of the volume and scope of studies available, and particularly useful for identifying emerging evidence when the research questions are not yet clearly defined. Scoping reviews are typically used when the topic is broad and there is a large volume of literature available; the research question is still evolving or not well-defined; there is a need to identify the key themes and concepts related to the topic; the goal is to provide an overview of the existing evidence rather than a comprehensive evaluation of individual studies; and/or there is limited time or resources available for a full systematic review. In fact, scoping reviews can help to guide the design of more specific and detailed systematic reviews by providing an understanding of the current state of knowledge on a topic [38]. As EMM is an emerging practice, a scoping review was judged as most suitable to identify and map the key concepts, ideas, and gaps in the existing literature [38]. We followed the Systematic Reviews and Meta-Analysis (PRISMA) guidelines for the reporting of scoping literature reviews [39]. In the scope of this review, we defined EMM vehicles as small-sized, electrically powered vehicles operating at speeds of up to a maximum of 25 km/h.

### Identifying Relevant Studies

This review derives from a larger, primary scoping exercise that identified the determinants of EMM use from a public health perspective, but also with the objective to gather all available research regarding health and safety impacts derived from the use of EMM including physical activity, noise and air pollution, safety risk, social cohesion, accessibility, and more. For this primary review, queries were carried out according to a systematic search strategy, using a combination of keywords covering EMM (i.e. the vehicles) AND health and safety pathways and mechanisms AND health, safety, and well-being outcomes (see an example in Table 1). The pathway and mechanism categories analysed were the following: *air pollution*, *noise, thermal comfort, route choice and natural outdoor environments, physical activity, safety and crash risk, trip purpose and motivation, accessibility and connectivity, infrastructure and management, regulation and compliance, use and behavior, Covid-19 and future trends.* We used the AND Boolean operator to create the final queries (shown in Table 1). The search was conducted in four different databases: PubMed, Web of Science (WoS), Scopus, and Transport Research International Documentation (TRID), to cover and reach all the topical dimensions: Web of Science and Scopus provide a multidisciplinary body of literature, PubMed covers biomedical and health sciences, and TRID that covers transportation sciences. Prior to the present research stage, to our knowledge, researchers reached a consensus on predefined umbrella health and safety pathway categories, that were thought to be relevant in the discussion on health and safety impacts of EMM [40].

**Table 1:** Selected keywords for the primary literature search by health pathway category

Keywords				
EMM		Health pathway (example: air pollution)		Health and well-being outcome
*electric micromobility OR e-micromobility OR electric two-wheeler OR electrification OR electric transport OR e-bike OR ebike OR electric bike OR electric bicycle OR e-bicycle OR e-cycling OR pedelec OR electric pedelec OR e-pedelec OR pedelec mobility OR electric scooter OR e-scooter OR electric kick-scooter OR electric motorbike OR e-motorbike OR electric motorcycle OR e-motorcycle OR electric moped scooter OR electric moped OR e-moped OR Segway OR e-skateboard OR electric skateboard OR e-longboard OR electric longboard OR hoverboard*	AND	*air pollution OR contamination OR greenhouse gases OR GHG OR dioxide nitrogen OR NO2 OR particulate matter OR PM10 OR PM2.5 OR sulphur dioxide OR SO2 OR ozone OR O3 OR lead OR Pb OR methane OR CH4 OR carbon dioxide OR CO2 OR carbon emissions OR carbon footprint*	AND	*health OR health effect OR health impact OR acute OR chronic OR well-being OR health impact assessment OR health impact evaluation OR disease OR disability OR morbidity OR mortality OR disability-adjusted life year OR DALY OR years of life lost OR YLL OR years lived with disability OR YLD OR quality-adjusted life-year OR QALY OR health burden OR burden of disease OR mental health OR quality of life OR life satisfaction OR life expectancy OR happiness OR depression OR anxiety OR dementia OR physical health OR cardiovascular disease OR respiratory disease OR cancer OR overweight OR obesity OR annoyance OR sleep disturbance OR injury OR fatality OR accident OR incident OR severity OR fall OR crash OR hospitalisation OR emergency room visit OR health cost OR productivity loss OR work absence*

**Fig. 1:**
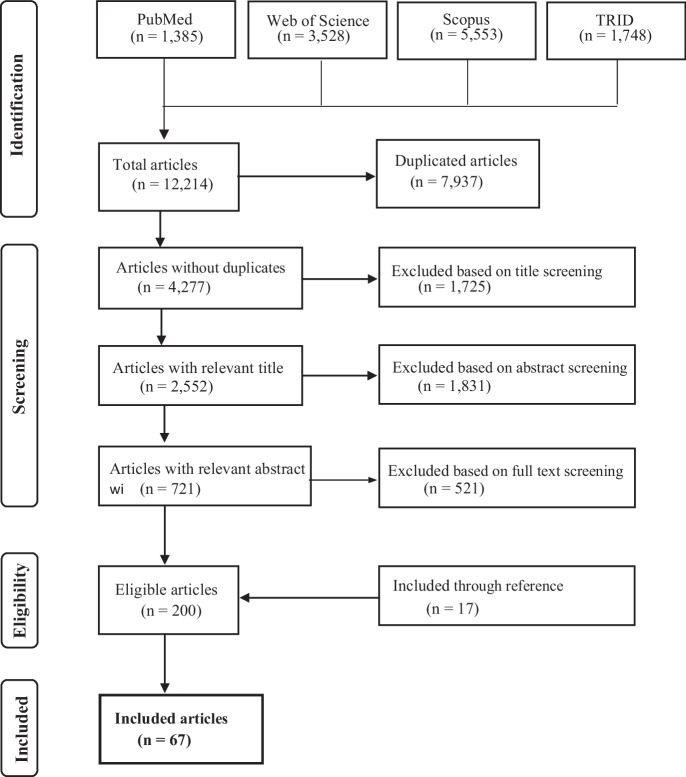
PRISMA flow diagram

All searches were limited to the English language, and to articles published between 2010 and 2021. All types of study designs were included: scoping review, systematic review, meta-analysis, ecological, longitudinal, cross-sectional, case–control, intervention, and observational. For review inclusion, the studies had to cover determinants of EMM use and a health or safety pathway or mechanism of EMM linking to human health. Searches in all four databases resulted in a total of 12,214 hits, as shown in Table 2 and Fig. 1.

**Table 2:** Search strategy database results

Database	Focus	Publication date	Hits
PubMed	Biomedical and health sciences	2010–2021	1385
Web of Science (WoS)	Multidisciplinary	2010–2021	3528
Scopus	Multidisciplinary	2010–2021	5553
Transport Research International Documentation (TRID)	Transportation science	2010–2021	1748
Total hits			12,214

Only studies covering EMM vehicles that met the definition provided above were included (i.e. light vehicles, powered by electricity, not exceeding a speed of 25 km/h). Therefore, e-mopeds, e-motorcycles, and speed-pedelecs were not included, as they likely exceed the speed definition. The scoping review is based on the international literature if authors of papers reviewed have judged discussed concepts to be universally relevant in the discussion on EMM determinants, irrespective of geographical context. However, the presented case studies were selected only for European settings, as the aim of this study is to reflect the current European EMM landscape, and it was thought that EMM determinants might vary in other parts of the world in accordance with local regulations, transport systems, user needs, behaviours, and experiences.

### Study Selection

Regarding the current scoping review on the determinants of EMM, of all the articles identified as eligible, we only focused on the publications dealing with determinants. A total of 67 articles were included for analysis, covering the enablers, motivations, barriers, and deterrents. We decided to classify the determinants between contextual enablers and barriers that refer to factors that operate at a larger or more macro level and that are related to the existing legal frameworks, transport system provisions, availability of dedicated infrastructure, technological enablers, and other wider, contextual factors (e.g. topography, climate, etc.); and personal motivations and deterrents, that are those relating to individuals’ preferences and intrinsic behaviours and depend on personal factors such as age, income, occupation, and lifestyle, including convenience, cost, time, health, environmental concerns, and social status.

All identified records were uploaded to the Mendeley references management software (https://www.mendeley.com). Duplicate publications were removed, and title and abstract screening were conducted. Publications meeting the established inclusion criteria were selected for full-text screening. Full texts were sourced, and full text screening was conducted. A descriptive analysis was carried out to gather information about the selected studies including their publication year, location, methodology, and outcomes. The main outcomes related to the specific topic of the review were identified and listed. This process was done by two reviewers, AB and NM, and a narrative summary was given for each outcome. The significance of the findings in relation to the research question and implications for research, policy, and practice were discussed, highlighting any evidence gaps and important priorities. All steps were carried out independently by the two researchers (AB, NM). Any discrepancies were resolved by consensus.

## Results

### Articles Retrieved

As the result of our search strategy for our primary review, and after the removal of duplicates, 4277 articles were title-screened, 2552 articles were abstract-screened, and 721 articles were full-text-screened (Fig. 1). After the full-text screening, 200 articles qualified for review inclusion. Additional cross-reading and reference screening added another 17 articles. Finally, 217 articles were included in the larger, primary scoping review, covering the landscape of EMM determinants and health and safety pathways. For the analysis presented here, of these 217 articles, 67 were identified to be specifically related to the determinants of EMM usage (i.e. in the European Region).

### Articles Characteristics

Of the 67 articles included in this scoping review, 85% (*n* = 57) were peer-reviewed research articles, and 15% (*n* = 10) were grey literature. Most of the peer-reviewed research was published in transport and mobility-related journals (47%), as well as in health and environmental science journals (32%). The grey literature contained eight reports and one project presentation. Regarding the time of publication, there was a steep increase in research on EMM since 2018, and 76% (*n* = 51) of the articles were published between 2018 and 2021. Of the total number of 67 publications, 76% (*n* = 51) presented an observational/cross-sectional research design, while 24% (*n* = 16) were literature reviews.

#### Observational Studies

Geographically, a total of 65% (*n* = 33) of articles originated from European research centres, predominantly in Norway, Poland, Netherlands, Portugal, the United Kingdom, Denmark, and Belgium. Of the remaining articles, 21% (*n* = 11) were from North America, 6% (*n* = 3) China, and 8% (*n* = 4) other countries including Singapore, New Zealand, Israel, and Saudi Arabia.

In terms of vehicle type, 71% (*n* = 36) of articles focused on e-bikes, and 27% (*n* = 14) focused on e-scooters. Only one study included both vehicles simultaneously. Moreover, 43% (*n* = 22) of articles focused on privately owned vehicles, while 41% (*n* = 21) explored EMM sharing systems and shared vehicles, and finally 16% (*n* = 8) included both privately owned and shared EMMs in their analyses.

Study populations and sample sizes were heterogeneous across the studies. Empirical studies, including focus groups and interview methods, consisted of sample sizes of 8 to 65 individuals, while studies using survey methods included responses of 200 to more than 100,000 individuals. Some studies targeted the entire population to study the populations’ mobility behaviours, and/or their willingness to adopt EMM, while others focused on the users of the transport mode being studied, or on specific target groups such as students, employees, older people, parents with children, inactive/sedentary people, or car owners. Additionally, there were studies comparing EMM users and non-users.

#### Literature Reviews

Apart from the observational studies, this scoping review also included literature reviews. 25% (*n* = 4) of reviews focused on e-bikes, while 31% (*n* = 5) focused on e-scooters. The remaining 44% of reviews (*n* = 7) included EMM modes in general, by using different terminologies such as electric two-wheelers, electric personal transportation devices (e-PTDs), and electric personal mobility vehicles (e-PMVs).

### E-micro-mobility determinants

This section lays out the determinants of EMM identified across the included studies. Table 3 and Table 4 summarise the contextual enablers and barriers, as well as individuals’ motivations for and deterrents against engaging in EMM use and list determinants according to commonness of reporting (i.e. most to least commonly reported).

**Table 3:**
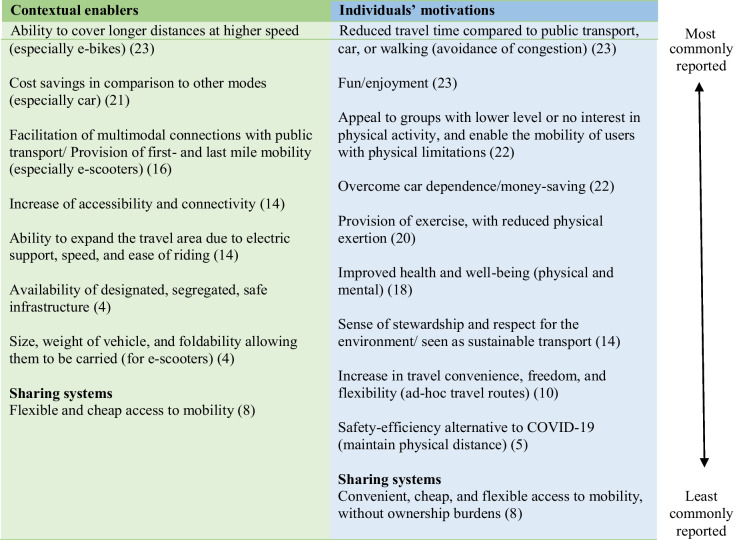
Contextual enablers and individuals’ motivations to engage in e-micro-mobility use, (the number in brackets represents the number of studies reporting that specific enabler)

**Table 4:**
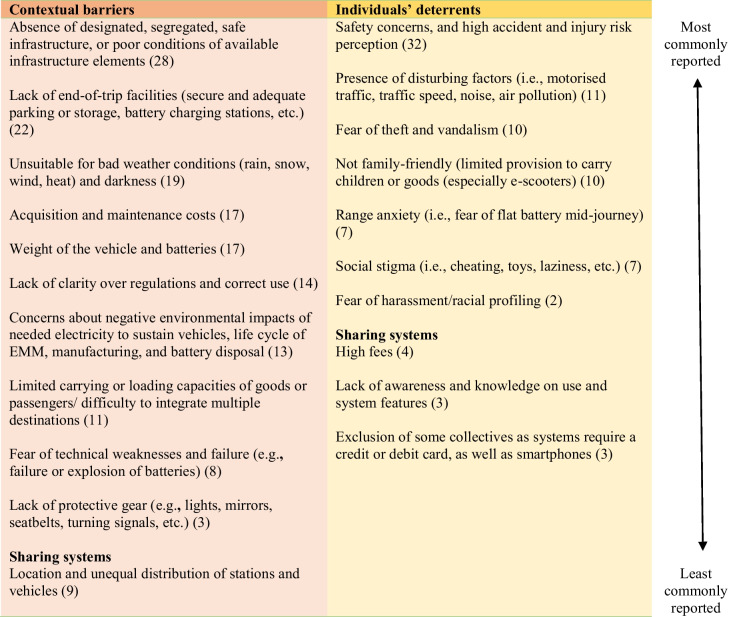
Contextual barriers and individuals’ deterrents to engaging in e-micro-mobility use, (the number in brackets represents the number of studies reporting that specific barrier)

#### Contextual Enablers for E-micro-mobility Use

##### Expanding Accessibility

EMM was found to offer a relatively cheap and fast way to move around [18, 19], expanding the area in which riders can travel easily without a car (or a driving licence), thereby, potentially increasing accessibility and connectivity for vulnerable population groups [14]. For instance, e-bikes offer the ability to cover longer distances at a higher speed, compared to conventional cycling or walking [24, 41–46]. An important consideration of EMM modes is the provision of first- and last-mile mobility [47] and, therefore, also the facilitation of multimodal connections with public transport [33, 48–50]. If vehicles are lightweight, foldable, and can be carried (i.e. especially e-scooters), they can be easily combined with, and taken on, public transport [33, 37].

##### Infrastructure: a Key Element to Foster Usage

Additionally, the provision of designated, segregated, and safe infrastructure for EMM use was repeatedly mentioned as a crucial element with which to foster and maintain its usage [45, 51]. In terms of EMM sharing systems, they were reported to offer a flexible, relatively cheap access to mobility, and to add to the diversity in transport opportunities [52].

#### Personal Motivations for E-micro-mobility Use

##### Convenience, Freedom, and Flexibility

According to the available evidence, various personal motivations for EMM use exist. Perceived increased travel convenience, freedom, flexibility, and overcoming car dependence [20, 21, 53] were found to be important to users. EMM allows the establishment of ad hoc, flexible, and more direct travel routes [54], which also contributes to overcoming car dependence, while avoiding traffic congestion [24, 25, 27, 55]. Reduced travel time, in comparison to public transport, car use or walking, factoring in waiting times for public transport, travelling during rush hour, and time spent looking for car parking, was also identified as a motivation for EMM use [16, 24, 26, 27, 35, 41, 56–58]. Potential travel time savings also relate to easy parking and storage (especially for e-scooters) [24, 34, 37]. Potential economic savings, especially in comparison to car use, were another reported motivator for adopting EMM [16, 23, 27, 59].

##### Physical Activity and Mobility for Users with Limitations

EMM also offers the benefits of performing some type of physical activity, without leading to physical exhaustion, assessed as especially important when riding uphill. In this sense, reduced sweating and need of having to shower, were identified as important motivators [12, 23–25, 41, 42, 46, 53, 58]. In fact, studies found that e-bikes enable travelling longer distances and reducing barriers regarding for people who do not engage in active transport, such as low perceived or actual fitness levels, hills, sweating, fatigue, and weather conditions [43, 44, 56, 60, 61]. Some studies noted that e-bikes strongly appeal to groups with lower levels of or no interest in physical activity, indicating that e-bikes can attract new user groups who might find a regular bike to be a non-alternative [37, 53]. Bourne et al. [36], for instance, reported older adults as being motivated to ride e-bikes, due to the physical activity provisions and associated health benefits. Additionally, EMM was reported to enable mobility for users with physical limitations [12, 21, 25]. In comparison to conventional active transport, EMM was perceived to be more suitable for everyday use when physically tired, dressed in formal attire, and carrying personal effects [43, 62].

##### Environmental Stewardship, Outdoor Experience, and Well-being

Other EMM motivators included a sense of stewardship and respect for the environment [23, 27], and the perception of using sustainable modes due to running on electricity [54]. Technology interest and innovativeness were also presented as inherent motivators to adopt EMM use. Felix et al. [63] stated that e-bikes can be perceived as “trendy” when compared to conventional bikes, which can be an additional motivator to engage in e-cycling.

Inhaling “fresh air” and being outdoors [24, 64]; fun, enjoyability, enhanced human experience [16, 21, 24, 25, 57]; “feeling like a little kid again” [55]; curiosity and fascination [31]; and the general contribution to increased well-being [24, 61], were all found to play positive roles, and strengthened the willingness to adopt and use EMM.

The more recent studies that were conducted in the context of the COVID-19 pandemic found EMM to be perceived as a safe alternative with respect to COVID-19 infection risk. EMM allows people to maintain physical and social distancing, at a time when individuals are/were hesitant to use public transport for fear of potential contamination [28, 49, 59, 61, 65]. Continued appreciation of transport that ensures physical distancing and privacy was found to be important in the post-pandemic period [59, 66].

##### Convenient, Easy, and Affordable Sharing Systems

Finally, with respect to sharing systems, users were found to appreciate convenient, easy, relatively cheap, and widespread access to mobility, without the burdens of vehicle ownership. Not having to buy, care for, maintain, and park/store the e-micro vehicle, but fully benefit from access to one, can be a motivator for EMM use [33, 66]. EMM sharing systems were found to be potential competitors for car and moped sharing systems, holding some significant advantages against these modes, such as not requiring a driving licence or, possibly, an age threshold [33].

#### Contextual Barriers for E-micro-mobility Use

##### Lack of Appropriate Infrastructure, Costs, and Limited Capacities

The absence of appropriate infrastructure, for example designated and segregated, and poor conditions of available infrastructure elements (e.g. uneven pavement, gravel) were important barriers to EMM use [12, 24, 27–29, 31, 36, 51, 53]. Likewise, the general lack of end-of-trip facilities (e.g. secure and adequate parking or storage, charging stations) has been noted as an important infrastructure-related deterrent for EMM ownership [36].

Moreover, vehicle acquisition and maintenance costs were generally found as deterrents [12, 24, 33, 34, 46]. Limited carrying or loading capacities of goods or passengers (e.g. children), in comparison to car use, can be barriers, especially when needing to combine several activities in one journey [12, 21, 24, 25, 28, 35, 41]. Particularly, private e-bikes were found to not be the best vehicle for multimodality purposes, as their size and weight make it difficult to swiftly hop off, when disembarking from trains and buses [35, 36, 45]. In addition, the weight of the vehicles, the batteries, and the general fear of technical issues or eventual failures are important concerns too [12, 24, 45].

##### Environmental and Weather Concerns, and Legal Framework

There also appear to be some worries about the electricity grid capacity that is needed to sustain an e-vehicle fleet [67], and their resulting emissions and environmental impacts [45]. Concerns regarding the life cycle assessment of the production and discarding of e-scooters and their batteries are considered by some EMM users as negative environmental impacts [54]. Additionally, weather conditions of rain, wind, snow, cold, or heat were also found to be potential barriers [2, 12, 24, 28, 35, 41], as well as travelling in the dark [67]. Finally, the lack of clarity of the existing legal framework and the correct use of vehicles were also mentioned as important barriers [37].

##### Accessibility, Availability, and Quality Concerns of Sharing Systems

Regarding sharing systems, accessibility to locations of docking-system stations, and the availability and unequal distribution of non-docked vehicles across the city, were identified as further barriers [29, 30, 33, 68]. Among French survey respondents, almost 25% stated that they gave up renting e-scooters because none were available nearby [21]. Vehicle safety and quality concerns of sharing system vehicles were identified as issues [68]. According to Kwiatkowski et al. [69], their study results revealed that, in cities with generally low levels of conventional cycling, the availability of an e-bike sharing system would probably not be a sufficient incentive to start cycling as a means of transport.

#### Personal Deterrents for E-micro-mobility Use

##### Safety and Security Concerns

The most important personal deterrents to engaging in EMM use were traffic safety concerns, and the increased accident and injury risk perception [12, 24, 27–30, 35, 51, 53, 68]. The presence of motorised traffic, traffic speed, and noise were perceived deterrents, as well as air pollution concerns [29, 32, 51]. Fear of theft and vandalism, including reservations about secure and appropriate public parking, was also mentioned in several case studies [12, 24, 35, 36, 45].

##### Perceived Social Stigma

Another personal barrier frequently found in EMM studies, is the existence of a perceived social stigma (i.e. social shaming) that can be linked with e-bike use. E-bikes are often (falsely) perceived as a form of “cheating” and not viewed as real bikes by cycling enthusiasts [41, 70]. Similarly, EMM vehicles are often perceived as “toys”, and their riders are deemed “lazy”, “overweight”, or “cheating” [12, 35, 37, 45, 71]. In the case of e-bikes, users are perceived as “old” [69], and these bikes are not yet accepted as transport modes by certain collectives [54]. Edge et al. [35] speculated that if e-bikes resembled conventional bikes, they would potentially get less scrutiny.

##### Shared Mobility Programmes: Addressing Accessibility and Affordability

There are a series of personal barriers related to sharing system programmes. Current non-users expressed a general lack of awareness on how to access or use these systems, which leads to being intimidated to figure out how they work or how to gain access to the system [29]. E-bike sharing systems were identified as not very family-friendly, with bikes being designed for adults only, and no provision for carrying children [29, 33, 72]. Limited distribution and accessibility of sharing stations/vehicles, and high access fees and user costs were reported as barriers [30], together with requiring a credit or debit card as a point of entry to the system, as well as a smartphone to access a supportive application. These barriers can lead to exclusion of socioeconomically disadvantaged or elderly people [21, 29], who do not possess the required resources or know-how to access sharing systems.

## Discussion and Conclusions

Based on a scoping review, we explored the landscape of the EMM literature and identified the determinants of EMM use in European settings. These determinants were further classified into either contextual/personal enablers or contextual/personal barriers. Our findings suggest a wide array of determinants that demonstrate the complexity and diversity of factors influencing the emergence, adoption, and sustained use of these new modes of transport in European cities.

### What Determines E-micro-mobility Use?

Regarding contextual enablers, EMM was found to offer a relatively cheap, flexible, ad hoc, and fast way to move within urban areas, expanding the area riders can easily travel without a car or a driving licence, thereby increasing accessibility and connectivity within cities. Although to date, findings on EMM being a first- and last-mile solution are mixed [16, 23, 32], users seem to appreciate the convenience of vehicles that are lightweight, foldable, and can be carried on public transport. Moreover, the provision of dedicated infrastructure for EMM appears to be a crucial element, with which to foster and maintain safe usage. Our findings highlight the lack of appropriate infrastructure and end-of-trip facilities as the main contextual barrier.

Considering personal motivations to adopt EMM, users value convenience, reduced travel times, low cost, low physical activity requirements, environmental sustainability, component innovation, and the thrill and enjoyment of riding. Regarding convenience, users seem to value freedom, and flexibility in deciding on travel routes and planning, reducing car dependence, congestion, and other car-related inconveniences. When factoring in waiting times for public transport, rush hour traffic, and time spent looking for car parking spaces, EMM was assessed to be an attractive time-saving competitor to other modes of transport. Economic savings seems to also be an important personal argument for why people might engage in EMM use, especially when compared to car use. However, high vehicle acquisition and maintenance costs were also reported as a contextual barrier when referring to privately owned EMM vehicles.

Physical activity was not found to be a significant factor in attracting new EMM users. Rather, it was the lower physical activity levels that are required to operate EMM which attracted users, especially in the case of e-bikes. EMM modes appear to be appealing for people with no or little interest in the physical activity component of transportation. Most importantly, EMM seems to be more suitable for everyday use in adverse conditions (cold weather, wearing formal clothing, physical tiredness) than other traditional active transport modes, which would indicate a growth potential for EMM, in some geographies and within a wider variety of social groups with different travel needs. Contrarily, some studies suggested that EMM use was weather dependent, and not particularly suited to the usual conditions of rain, wind, cold, heat, or darkness.

Regarding personal motivations, reviewed studies have found how the environmental perception that is linked to these vehicles favours their adoption, particularly among the more environmentally aware groups of younger people. This generalised perception of environmental friendliness might clash with more recent sustainability analyses which conclude that the rise of EMM in cities is causing an actual increase in emissions [37, 73]. Other appealing factors mentioned less frequently include the technological and innovation components of EMM and the enjoyability and personal experience of riding. Interestingly, the proper device technology was also identified as a barrier, including the increased weight of the vehicles and their batteries, and concerns about potential technical failures (e.g. battery explosion anxiety).

In relation to other contextual barriers, apart from the absence of satisfactory infrastructure, the limited capacity of EMM to cover all terrains and trip characteristics was also mentioned, together with the reduced carrying or loading capacities of goods and passengers. The most important personal deterrents to engaging in EMM use concerned safety and the increased accident and injury risk perception. The presence of disruptive factors such as dense motorised traffic, traffic speed, noise, and air pollution, together with the fear of theft and vandalism, were further identified as important deterrents.

Lastly, for the specific case of both docked and free-floating sharing systems, reviewed studies highlighted the reduced burdens of ownership and the convenience linked to the low levels of care and maintenance that are required as the main motivators to use these systems. Yet, docking locations, vehicle availability, and unequal vehicle distribution across the city were identified as the main contextual barriers. Likewise, personal deterrents were technological problems, lack of awareness of the features of the system, difficulty in determining how to use the system, as well as system cost and high fees. Moreover, these shared systems are sometimes perceived as excluding the socioeconomically disadvantaged or elderly people as the service requires a credit or debit card as a point of entry, as well as possession of a smartphone.

### Implications for Policy and Practice

Our findings suggest that EMM has the potential to provide mobility opportunities, diversify transport, and possibly even provide environmental and health benefits if they are properly managed. The introduction of e-bikes, and e-scooters, may have positive outcomes if they are well integrated into the existing (public) transport structures. EMM needs to be integrated well into the existing (public) transport system, facilitate first- and last-mile mobility, and particularly encourage mode shifts from private motorised transport (i.e. cars, motorcycles), to reap the largest environmental and health benefits. To do so, it is necessary to increase the availability and accessibility of EMM options, such as through the expansion of charging infrastructure and the creation of dedicated lanes. Regarding parking and storage, safe, secure, and vehicle-appropriate parking spaces are needed at different locations (e.g. work, public transport stops, kindergartens, shopping centres, other points of interest), including overnight parking. Additionally, measures are required to improve the affordability of privately owned devices, such as subsidies or reduced tariffs. EMM use is thought to provide a range of benefits at the individual level, such as perceived increased well-being, enjoyability, flexibility and freedom, and money- and time-savings that can be capitalised on.

For EMM to become a viable and safe mobility alternative for increasing numbers of people around Europe, a clear legal framework that prevents conflicts with other road users is needed. This literature review has identified traffic safety concerns as the main barrier for adoption among potential users. Authorities should not only address this issue, but also create and disseminate clear rules and guidance regarding which infrastructures EMM is supposed to use (e.g. this would work best if they are designated and segregated), and which other safety and public order requirements are in place (e.g. parking requirements, maximum speeds allowed, minimum user age, safety, and visibility gear). Moreover, clear enforcement of established rules is also necessary to avoid conflicts and ensure a safe co-existence, and appropriate public space allocation and usage (e.g. avoidance of “cluttering”).

Several measures could be implemented to make the use of EMM more socially accessible. In the first instance, design could focus on easing access and use for certain collectives (e.g. women, elderly, cargo, and delivery), thus reducing the vehicle weight and increasing stability and manoeuvrability. Vehicles could be made generally more cargo- and family-friendly by allowing installations to carry goods and child seats. Sharing systems could offer alternative access paths, that do not necessarily involve access to the latest smartphone technology or using debit and credit cards. This would require an alternative payment system with a physical infrastructure such as kiosks or rental stations to pay by cash. Also, pre-paid cards could be accepted so they can be loaded with funds prior to the rental. These measures would help reduce discrimination against certain communities. Our review has also found that there is a strong identification of EMM as a sustainable alternative to traditional transport modes. This contrasts heavily with recent literature pointing to the contrary, suggesting that for instance when EMM replaces other active forms of transport such as walking or biking it would be causing a damage to the environment by increasing emissions, while there exist environmental challenges posed by the manufacture and disposal of batteries, if the whole life cycle of these vehicles is examined [35, 67, 74–76]. These authors highlight the need to raise awareness about the real implications of EMM and its impact on the environment. Hence, it seems that EMM environmental benefits may be overestimated by some individuals, as this information is not widely known by its current or potential users.

It is important to note that all these policy recommendations will also depend on the different levels of government and stakeholders that may be involved in implementing policy changes related to EMM, as well as the potential barriers and facilitators to implementing policy changes. At the local level, decisions related to EMM may include the installation of charging infrastructure and the creation of dedicated lanes. Local governments may also be responsible for enforcing safety regulations and monitoring the use of EMM in their jurisdictions. At the state level, decisions may include regulations related to the operation and licencing of EMM companies and operators, as well as the development of policies to promote their use. At the country level, decisions may include regulations related to safety and operation, as well as the development of national transportation policies that incorporate EMM and corresponding regulatory frameworks. In any case, the level of government that is responsible for making decisions related to EMM may vary depending on the specific issue and the country or region. The same applies to the success of these potential measures. Successes could vary across different towns and cities, as different jurisdictions may have different needs and resources. Success in certain locations than others depends on factors such as population density, existing transportation infrastructure, and cultural attitudes towards EMM. Therefore, it is crucial to consider the local context and the specific needs of different communities when developing and implementing policy recommendations related to EMM. Finally, additional research, discussion, and communication are essential among authorities, researchers, and practitioners to improve current mobility systems, make evidence-based decisions, and potentiate positive (environmental and health) impacts of EMM use, while mitigating negative effects.

## Abbreviations

EMM: Electric micro-mobility
e-PMVs: Electric personal mobility vehicles
e-PTDs: Electric personal transportation devices
TRID: Transportation Research International Documentation
WoS: Web of Science
